# Multi-Task Network Representation Learning

**DOI:** 10.3389/fnins.2020.00001

**Published:** 2020-01-23

**Authors:** Yu Xie, Peixuan Jin, Maoguo Gong, Chen Zhang, Bin Yu

**Affiliations:** ^1^School of Computer Science and Technology, Xidian University, Xi'an, China; ^2^Key Laboratory of Intelligent Perception and Image Understanding of Ministry of Education, School of Electronic Engineering, Xidian University, Xi'an, China

**Keywords:** multi-task learning, representation learning, node classification, link prediction, graph neural network

## Abstract

Networks, such as social networks, biochemical networks, and protein-protein interaction networks are ubiquitous in the real world. Network representation learning aims to embed nodes in a network as low-dimensional, dense, real-valued vectors, and facilitate downstream network analysis. The existing embedding methods commonly endeavor to capture structure information in a network, but lack of consideration of subsequent tasks and synergies between these tasks, which are of equal importance for learning desirable network representations. To address this issue, we propose a novel multi-task network representation learning (MTNRL) framework, which is end-to-end and more effective for underlying tasks. The original network and the incomplete network share a unified embedding layer followed by node classification and link prediction tasks that simultaneously perform on the embedding vectors. By optimizing the multi-task loss function, our framework jointly learns task-oriented embedding representations for each node. Besides, our framework is suitable for all network embedding methods, and the experiment results on several benchmark datasets demonstrate the effectiveness of the proposed framework compared with state-of-the-art methods.

## 1. Introduction

Networks are ubiquitous in the real world, and can be organized in the form of graphs where nodes represent various objects and edges represent relationships between objects. For examples, in a protein-protein interaction network (Wang et al., 2019), the physical interactions among proteins constitute the networks of protein complexes where each individual protein is an independent node and the interaction represents an edge. In medical practice (Litjens et al., 2017), analyzing protein-protein networks can gain new insights into biochemical cascades and guide the discovery of putative protein targets of therapeutic interest. For efficiently mining these complex networks, it is necessary to learn an informative and discriminative representation for each node in the complex network. Therefore, network representation learning (Cui et al., 2019), also known as graph embedding (Yan et al., 2005), has attracted a great deal of attention in recent years.

Existing network representation learning methods can be generally divided into two categories, including unsupervised and semi-supervised methods. Unsupervised network representation learning methods (Khosla et al., 2019), such as DeepWalk (Perozzi et al., 2014), node2vec (Grover and Leskovec, 2016), and GraphGAN (Wang et al., 2018), explore specific proximities and topological information in a complex network and optimize the carefully designed unsupervised loss for learning node representations, which can be used for subsequent node classification (Kazienko and Kajdanowicz, 2011) and link prediction (Liben-Nowell and Kleinberg, 2007; Lü and Zhou, 2011). Semi-supervised network representation learning methods (Li et al., 2017), such as GraphSAGE (Hamilton et al., 2017), GAT (Veličković et al., 2018), and so on, develop end-to-end graph neural network architectures for semi-supervised node classification based on the partial labeled nodes and other unlabeled nodes in hand. However, all of these methods are lack of adequate consideration for subsequent network analysis tasks. More specifically, unsupervised network representation learning methods inherently ignore the category attributes of nodes. Both unsupervised and semi-supervised network representation learning methods are not supervised by the link prediction task in the process of learning desirable node representations. The only existing work is that, Tran et al. presented a densely connected autoencoder architecture (Zhu et al., 2016), namely local neighborhood graph autoencoder (LoNGAE, αLoNGAE) (Tran, 2018), to learn a joint representation of both local graph structure and available external node features for the multi-task learning (Yu and Qiang, 2017) of node classification and link prediction. Nevertheless, it has poor scalability on general network embedding methods due to the use of autoencoder.

As a bridge between the graph structured network data and the underlying network analysis task, network representation learning algorithms should not only preserve the proximities and complex topological structure, but also learn high-quality node representations for enhancing the performance of relevant tasks. Fortunately, multi-task learning (MTL) is a standard paradigm that takes full advantage of the synergy among tasks to make multiple learning tasks promote each other (Yu and Qiang, 2017). In deep learning (LeCun et al., 2015), multi-task learning (Caruana, 1993) is usually implemented by sharing the soft or hard parameters of the hidden layer. Each task has its own parameters and models when sharing soft parameters. The distance between model parameters is regularized to encourage parameter similarity. Sharing the hard parameter is the most common method of multi-task learning on neural networks, which significantly reduces the risk of overfitting.

Inspired by this, we attempt to propose a universal multi-task network representation learning (MTNRL) framework, which can be implemented on general network embedding methods for link prediction and node classification. To enable the traditional network embedding methods to effectively learn multiple tasks synchronously, two different network analysis tasks share parameters of the feature extraction module and retain its own task-specific module in our framework. The shared feature extraction module is utilized for learning the latent low-dimensional representations of nodes in a complex network. The task-specific module takes the obtained node representations as input and incorporates the losses of node classification and link prediction tasks. Through jointly optimizing the overall losses, we can learn the desirable network representations and improve the classification or prediction results of different tasks. Besides, our proposed MTNRL framework has good universality and can be applied to almost all of the existing network representation learning approaches.

The main contributions of this paper are summarized as follows:

- We propose a novel multi-task network representation learning (MTNRL) framework, which simultaneously performs multiple tasks including node classification and link prediction by sharing the intermediate embedding representations of nodes.
- The proposed framework is implemented on state-of-the-art graph attention neural networks in detail for illustration.
- We conduct empirical evaluation on three datasets and the experimental results demonstrate that the proposed framework achieves similar or even better results than existing original network representation learning methods.

The rest of this paper is arranged as follows. We first summarize related works in section 2. Section 3 presents our proposed multi-task network representation learning framework for node classification and link prediction. Section 4 describes the experimental settings and results, while conclusions are discussed in section 5.

## 2. Related Work and Motivation

### 2.1. Network Representation Learning

Recently, network representation learning has attracted an increasing research attention in various fields. Existing network representation learning techniques can roughly be divided as unsupervised and semi-supervised. Given a complex network with all nodes being unlabeled, unsupervised methods learn node representations through optimizing the carefully designed objective to capture proximities and topology in the network graph, which can facilitate identifying the class labels for the nodes. Deepwalk (Perozzi et al., 2014) regards the sequence of nodes generated by random walk (Tong et al., 2006) as a sentence, the nodes in the sequence as words in the text, and obtains node representations through optimizing the Skip-Gram model (Lazaridou et al., 2015). LINE (Tang et al., 2015) characterizes the first-order proximity observed from the connections among nodes, and preserves the second-order proximity through calculating the number of common neighbors for two nodes without direct connection. Node2vec (Grover and Leskovec, 2016) extends the Deepwalk algorithm by introducing a pair of hyper-parameters for adding flexibility in exploring neighborhoods, and generates random walk sequences by breadth-first search (Beamer et al., 2013) and depth-first search (Barták, 2004).

Unsupervised learning begins with clustering and then characterization, while supervised learning is carried out simultaneously with classification and characterization. Semi-supervised learning is a classic paradigm of machine learning between supervised learning and unsupervised learning. In this paradigm, a small amount of labeled data and a large number of unlabeled data are used to train the learning model. In practice, it is arduous to obtain a great deal of labeled data and semi-supervised learning is capable of improving the performance of purely supervised learning algorithms through modeling the distribution of unlabeled data. Therefore, semi-supervised learning has received considerable attention in recent years. Semi-supervised learning methods utilize partial nodes being labeled and others remaining unlabeled to learn high-quality node representations supervised by partial nodes. For examples, graph convolution networks (GCN) (Kipf and Welling, 2017) generalizes the original convolutional neural networks on grid-like images to non-grid graphs through considering the localized first-order approximation of spectral graph convolutions for encoding graph structure and optimizing the cross-entropy loss over labeled node examples for semi-supervised node classification. Given a graph composed of instance nodes, Planetoid (Yang et al., 2016) presents a semi-supervised learning framework based on graph embeddings which can train an embedding for each instance to jointly predict the class label and the neighborhood context in the graph. This method has both transduction variables and induction variables. While in the inductive variant, the embeddings are defined as a parametric function of the feature vectors, so predictions can be made on instances not seen during training. GraphSAGE (Hamilton et al., 2017) is an inductive network representation learning framework that learns an embedding function for generating node representations through sampling a fixed-size set of neighbors of each node, and then performing a specific aggregator over neighboring nodes (such as the mean over all the sampled neighbors' feature vectors, or the result of feeding them through a recurrent neural network). Graph attention networks (GAT) (Veličković et al., 2018) operate on graph-structured data, leveraging masked self-attentional layers (Zhang et al., 2018) to address the shortcomings of prior methods based on graph convolutions. These methods are all implemented as a single task, but multi-task learning can be used to improve the performance of multiple tasks simultaneously.

### 2.2. Multi-Task Learning

Multi-task learning is a promising area of machine learning that leverages the useful information contained in multiple learning tasks to help learn each task more accurately. Multi-task learning is capable of learning more than one learning task simultaneously, because each task can take advantage of the knowledge of other related tasks. Traditional multi-task learning methods (Doersch and Zisserman, 2017) can be classified into many kinds, including multi-task supervised learning, multi-task unsupervised learning (Kim et al., 2017), and multi-task semi-supervised learning (Zhuang et al., 2015). Multi-task supervised learning implies that each task in multi-task learning is a supervised learning task, which models the function mapping from examples to labels. Different from the multi-task supervised learning with labeled examples, the training set of multi-task unsupervised learning only consists of unlabeled examples to mine the information contained in the dataset.

### 2.3. Motivation

In many practical applications, there is usually only a small amount of labeled graph data, because manual annotation wastes labor and time considerably (Navon and Goldschmidt, 2003). For example, in biology, the structure and function analysis of a protein network may take a long time, while large amounts of unlabeled data are easily available. Hence, semi-supervised learning methods are widely used to improve learning performance of graph analysis. Unfortunately, all of the aforementioned semi-supervised learning methods applied on graphs, such as GCN, GraphSAGE, and GAT only learn the latent node representations in a single-task oriented manner and lack consideration of the synergy among subsequent graph analytic tasks. In reality, tasks of node classification and link prediction usually share some common characteristics and can be conducted simultaneously for facilitating each other.

As far as we know, the only existing work is the local neighborhood graph autoencoder (LoNGAE, αLoNGAE), which implements the multi-task network representation learning based on a densely connected symmetrical autoencoder and is model dependent. The model utilizes the parameter sharing between encoders and decoders to learn expressive non-linear latent node representations from local graph neighborhoods. Motivated by this, we innovatively propose a general multi-task network representation learning (MTNRL) framework, which is model-agnostic and can be applied on arbitrary network representation models. It optimizes the losses of two tasks jointly to learn the desirable node representations followed by node classification and link prediction tasks that performed on the embedding vectors.

## 3. Methodology

In this section, we formally define the problems of network representation learning and multi-task learning. Then the proposed MTNRL framework and its implementation on graph attention networks are elaborated in detail.

### 3.1. Problem Formulation and Notations

A network is usually denoted as *G* = (*V, E*), where *V* = {*v*_1_, ⋯ , *v*_*n*_} represents a set of nodes and *n* is the number of nodes. $E={\left\{e_{i,j}\right\}}_{i,j=1}^{n}$ denotes the set of edges between any two nodes. Each edge *e*_*i,j*_ can be associated with a weight *a*_*i,j*_ ≥ 0, which is an element of the adjacency matrix *A* for the network *G*. In an unweighted graph, for nodes *v*_*i*_ and *v*_*j*_ not linked by an edge, *a*_*i,j*_ = 0, otherwise, *a*_*i,j*_ = 1. Formally, we define the following two problems closely related to our work.

Definition 1 (**Network representation learning**). Given a network *G* = (*V, E*), network representation learning aims to learn a function *f*:*V* → *R*^*n* × *d*^, that maps each node into a *d*-dimensional embedding space. Meanwhile, *d* is the dimension of latent representations and *d* ≪ *n*.

Definition 2 (**Multi-task learning**). Given multiple related learning tasks, the goal of multi-task learning is to improve the performance of each task by jointly learning these related tasks and mining the useful information contained in these tasks.

The main symbols used throughout this paper are listed in Table 1.

**Table 1:** Notations and their descriptions.

**Notations**	**Descriptions**
*G*	The given network
*V*	Set of nodes in the given network
*E*	Set of edges in the given network
*v*	A node *v* ∈ *V*
*e*_*i, j*_	An edge between nodes *v*_*i*_ and *v*_*j*_
*n*	Number of nodes in the given network
*c*	The number of class labels for nodes in *V*
*A*	The adjacency matrix of *G*
*d*	The dimension of learned node representations
*Z*	The initial feature matrix of nodes
*H*	The embedding representation matrix of nodes

### 3.2. Framework

Aiming to obtain the compact and expressive representation of a complex network, network representation learning is widely used in a variety of applications, including node classification, link predication, and so on.

As one of the most important application for network representation learning, node classification attempts to assign the predicted class label to each node in the network based on the patterns learnt from the partially labeled nodes. Intuitively, similar nodes in a complex network should have the same labels. The results of node classification are often used in recommendation systems and data mining systems. Because in these practical applications, nodes in a complex network are only partially labeled due to high labeling costs, and a large portion of vertices in networks do not have ground truth. According to the number of labels of each node in a network, node classification can be categorized into multi-class node classification and multi-label node classification. In multi-label node classification, each node may correspond multiple labels, while each node only has one label in multi-class node classification. Essentially, node classification based on existing network representation learning techniques typically consist of two stages: representation learning and node classification.

With the carefully designed network embedding algorithm, a network graph *G* can be taken as input to the embedding model *f* for learning the low-dimensional dense representation *H* in an unsupervised or semi-supervised manner, which is expressed as:

(1) $$\begin{array}{r} H=f\left(A,Z\right) \end{array}$$

*A* denotes the adjacency matrix of *G* and *Z* is the initial feature representation of nodes, which can be represented by nodes' feature property or other properties. For unsupervised network representation learning, the obtained node representations are then utilized to train a supervised classifier for node classification. Semi-supervised network representation learning directly trains a classifier well for classification while training the embedding model. With the well-trained classifier, we can infer the labels of the remaining nodes. The performance of node classification is reflected by the predicted accuracy for node labels. The loss function of node classification can be defined as follow:

(2) $$\begin{array}{r} L_{NC}=-\underset{v\in V_{L}}{\sum }\overset{c}{\underset{k\;=\;1}{\sum }}y_{v,k}*\log\left(P_{v,k}\right) \end{array}$$

where *V*_*L*_ is the set of labeled nodes and *c* denotes the number of class labels. *y*_*v,k*_ represents an indicator variable of node *v*, which is equal to 1 if node *v* belongs to class *k*, otherwise 0. *P*_*v*_ is the predicted probability vector of node *v* and can be calculated by $P_{v}=\text{softmax}\left(W^{T}h_{v}+b\right)$, in which *h*_*v*_ is the embedding representation of node *v*, *W* is the weight matrix, and *b* is the bias in the final fully connected layer.

Another fundamental application for network representation learning is link predication. Link prediction endeavors to predict the existing possibility of edges between two nodes in a network that are unobserved or missing by utilizing available network nodes and topological structure. In general, we randomly hide a portion of the existing links for simulation and use the left edges to train an unsupervised network embedding model. To seamlessly integrate the tasks of link prediction and node classification, we design a loss function for link prediction as:

(3) $$\begin{array}{r} L_{LP}=-\overset{n}{\underset{i\;=\;1}{\sum }}\overset{n}{\underset{j\;=\;1}{\sum }}\left[A_{i,j}*\log\left(S_{i,j}\right)+\left(1-A_{i,j}\right)*\log\left(1-S_{i,j}\right)\right] \end{array}$$

where *A*_*i,j*_ is an element of the adjacency matrix of a network *G* and *n* indicates the number of nodes. *S*_*i,j*_ = s(*h*_*i*_, *h*_*j*_) is a score of the predicted link between nodes *v*_*i*_ and *v*_*j*_, which can be calculated with the inner product or other similarity measure between embedding representations *h*_*i*_ and *h*_*j*_. A larger score usually implies that the two nodes may have a higher likelihood to be linked. With the loss in Equation (3), we can learn the structural representations for each node in the network graph and then utilize the obtained representations to predict the unobserved link.

**Figure 1:**
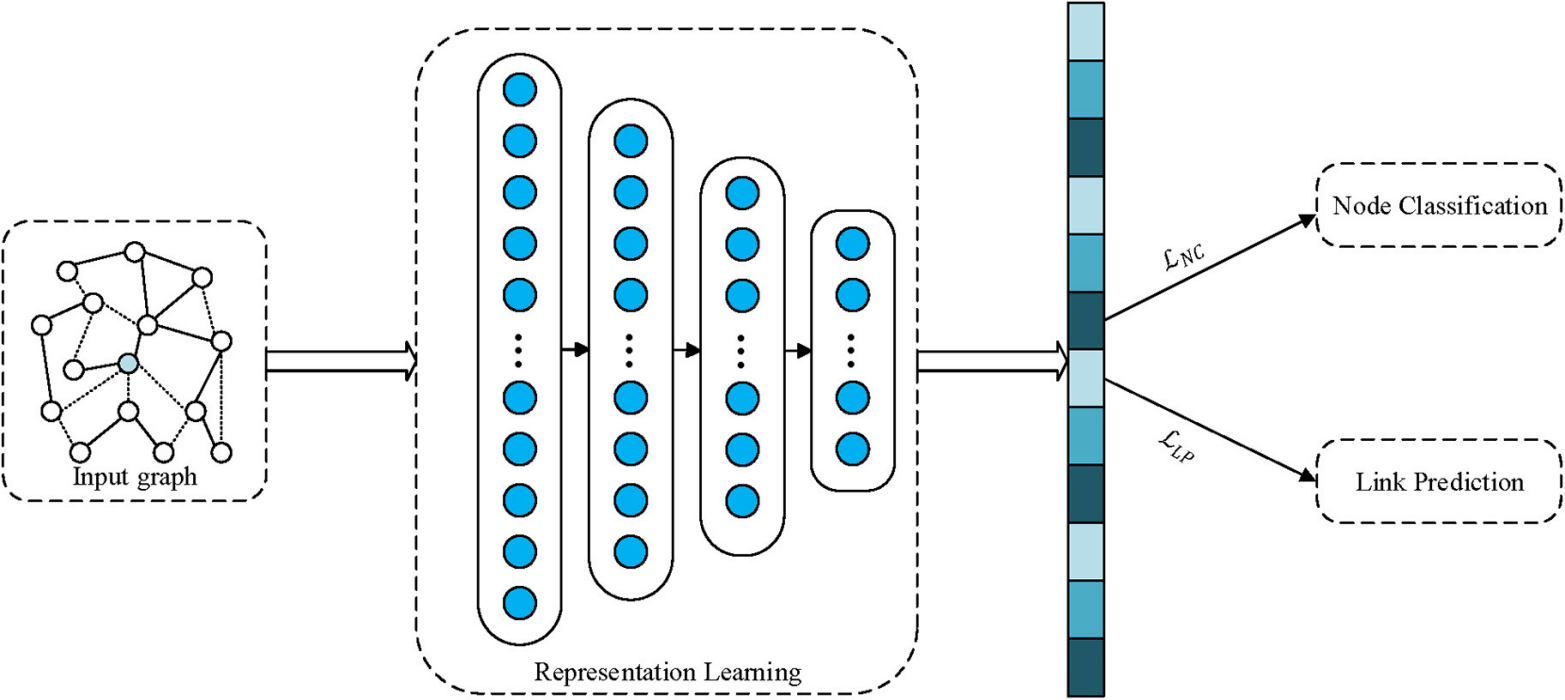
Graphical illustrations of our proposed multi-task network representation learning framework.

To benefit subsequent tasks of both node classification and link prediction, we learn informative and discriminative graph representations collaboratively supervised by these two tasks. More specifically, the overall loss function for multi-task network representation learning (MTNRL) can be formulated as:

(4) $$\begin{array}{r} L=L_{NC}+\alpha L_{LP} \end{array}$$

where α is a tradeoff factor for balancing losses of node classification and link prediction. For illustration, our MTNRL framework is shown in Figure 1. A network graph is taken as the input to a network representation learning model. By virtue of the network representation learning model for graph-structured data, the proximity and topological structure will be preserved in the embedding representations. Furthermore, we simultaneously perform node classification and link prediction tasks through optimizing the carefully designed multi-task loss function on the node representations obtained from the representation learning module. As a result, we jointly learn task-oriented embedding representations for each node, which are capable of improving the performance of a variety of graph analytics applications.

### 3.3. Implementation on Graph Attention Networks

Graph attention networks (GAT) (Veličković et al., 2018) introduce an attention-based architecture to learn the node-focused representations for node classification on graph-structured data. GAT is based on the classical neighbor aggregation schema for generating low-dimensional node representations and extends the pioneering graph convolutional networks through exploring the importance of different neighboring nodes. Based on the attention mechanism widely used in sequence-based tasks, GAT calculates an attention coefficient $e_{ij}=a\left(\text{W}{\vec{h}}_{i},\text{W}{\vec{h}}_{j}\right)$ for pairwise nodes. Suppose $\text{h}=\left\{{\vec{h}}_{1},{\vec{h}}_{2},\ldots ,{\vec{h}}_{N}\right\},{\vec{h}}_{i}\in {\mathbb{R}}^{F}$ is a set of node features used as the input to the attention layer, where *N* is the number of nodes, and *F* is the number of features for each node. A shared linear transformation, parameterized by a weight matrix, **W** ∈ ℝ^*F*′ × *F*^, is applied to every node. Then the shared attentional mechanism *a*:ℝ^*F*^′^^ × ℝ^*F*^′ → ℝ is utilized to calculate *e*_*ij*_. With the normalized attention coefficients $\alpha_{ij}={\mathrm{softmax}}_{j}\left(e_{ij}\right)=\frac{\exp\left(e_{ij}\right)}{\underset{k\in N_{i}}{\sum }\exp\left(e_{ik}\right)}$, we can pay different attention to the neighboring nodes when attending over its neighbors for generating the latent representation of each node. Therefore, the normalized attention coefficients are used to compute a linear combination of the features corresponding to them, to serve as the final output features for every node (after potentially applying a non-linear function σ): ${\vec{h}}_{i}^{\prime }=\sigma \left(\underset{j\in N_{i}}{\sum }\alpha_{ij}\text{W}{\vec{h}}_{j}\right)$, where ${\text{h}}^{\prime }=\left\{{\vec{h}}_{1}^{\prime },{\vec{h}}_{2}^{\prime },\ldots ,{\vec{h}}_{N}^{\prime }\right\},{\vec{h}}_{i}^{\prime }\in {\mathbb{R}}^{F^{\prime }}$ is a new set of node features produced by the attention layer. By optimizing the loss of semi-supervised node classification, GAT learns the representation of nodes. By stacking to multiple layers, a deep graph attention network can be constructed for capturing the high-order topological relationship among nodes in a graph.

**Figure 2:**
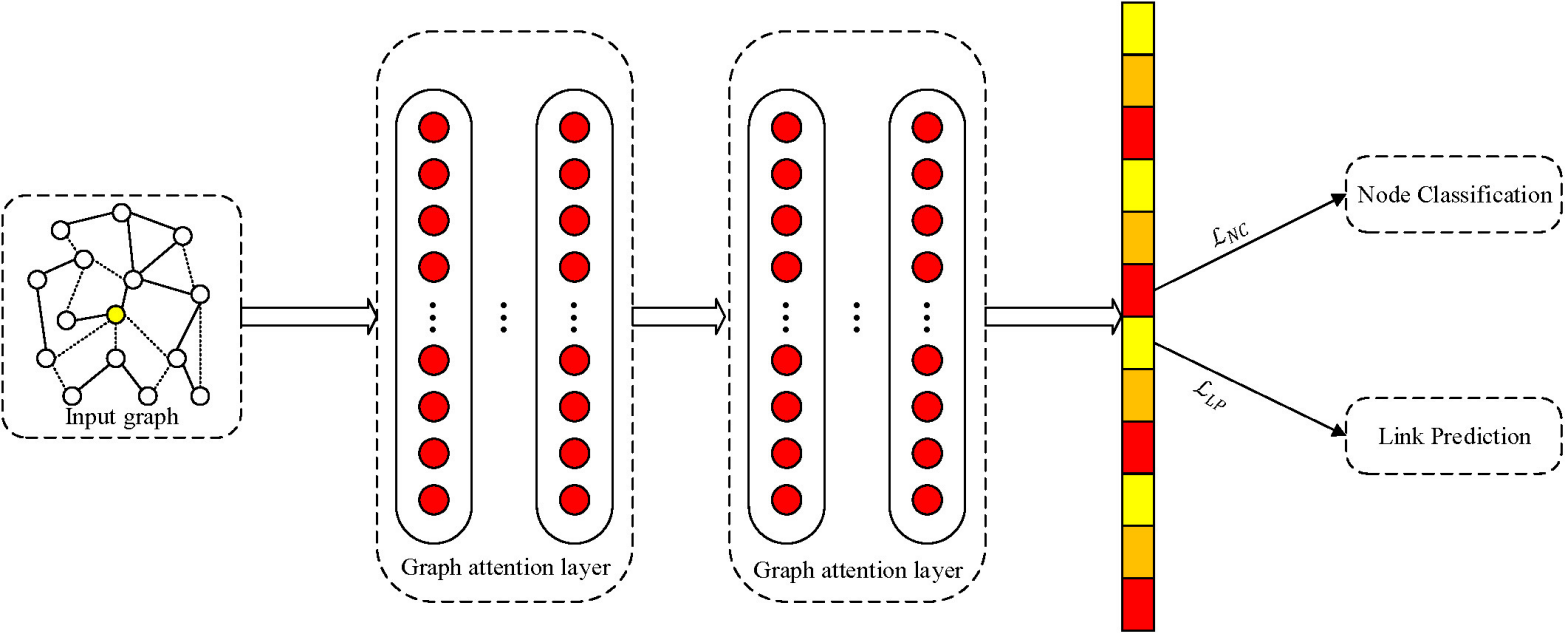
Schematic depiction of implementation of the proposed framework on graph attention networks.

The proposed MTNRL framework can be implemented on arbitrary network representation learning methods. In this subsection, we introduce an implementation of the MTNRL framework on graph attention networks (MT-GAT) as an example. The original graph attention networks adopt a two-layer GAT model for inductive learning, which can predict the labels of nodes in a semi-supervised manner based on the masked self-attention operated on graph-structured data. In our implementation of MT-GAT, node classification and link prediction tasks are predicted simultaneously. As shown in Figure 2, a network graph is taken as input to graph attention networks that can output compact embedding representations of nodes. Then we use the learned low-dimensional node representations for multi-task learning. In the MT-GAT, all parameters in the network except the softmax layer for node classification are shared. In this implementation, the loss function of node classification employs a negative log likelihood loss and the loss function of link prediction adopts a two-class cross entropy loss, which is in consistent with Equations (2) and (3).

### 3.4. Discussion

To further demonstrate that our MTNRL is a universal framework, we explain how it can be used in Graph Convolutional Networks (GCN) (Kipf and Welling, 2017). GCN is a classical convolutional neural network architecture applied to graph-structured data, which can explicitly characterize the first-order neighboring structure and be stacked to multiple layers for encoding high-order proximities in a network. The original GCN only optimizes the semi-supervised node classification loss for learning latent node representations. Under the proposed MTNRL framework, we can optimize the loss functions of both node classification and link prediction tasks at the same time. Through further assigning the proper weights to the losses of two tasks, we can complete the implementation of our MTNRL framework on GCN.

## 4. Experiment

We conduct the experimental evaluation of the proposed multi-task network representation learning framework on graph attention networks (MT-GAT), compared with state-of-the-art methods. This section first introduces the specifics of experimental datasets and several baselines. Then, we present the details of the implementation, followed by experimental results and analysis of different algorithms. Finally, we analyze the sensitivity of the hyperparameters.

### 4.1. Datasets

We adopt three benchmark citation network datasets for evaluation, including Cora, Citeseer, and Pubmed (Sen et al., 2008), whose detailed statistics are summarized in Table 2. For these citation networks, each paper is denoted as a node and the words of each paper are encoded as the features of nodes which is a vocabulary containing multiple words. Each node only corresponds a class label. The features of the paper consist of a string of binary codes, which indicate whether the paper contains this word.

- The Cora dataset consists of 2,708 papers from machine learning area and these papers are divided into the seven categories: Case Based, Genetic Algorithms, Neural Networks, Probabilistic Methods, Reinforcement Learning, Rule Learning, Theory. The citation network consists of 5,429 edges that represent citation relationships. The text information of each publication is encoded by a tf-idf vector of 1,433 dimensions indicating the importance of the corresponding words.
- The Citeseer dataset consists of 3,312 scientific publications from the CiteSeer web database, and are categorized into six classes: Agents, Artificial Intelligence, Data Base, Information Retrieval, Machine Language, and HCI. The citation network consists of 4,732 links. Each publication in the dataset is described by a 0/1-valued word vector indicating the absence/presence of the corresponding word from the dictionary. The dictionary consists of 3,703 unique words.
- The Pubmed dataset consists of 19,717 scientific publications from PubMed database pertaining to diabetes classified into three classes: Diabetes Mellitus Experimental, Diabetes Mellitus Type 1, Diabetes Mellitus Type 2. The citation network consists of 44,338 links. Each publication in the dataset form a dictionary which is made up of 500 unique words.

**Table 2:** Statistics of benchmark datasets used in our experiments.

**Datasets**	**Cora**	**Citeseer**	**Pubmed**
Nodes	2,708	3,327	19,717
Edges	5,429	4,732	44,338
Text feature dimension	1,433	3,703	500
Classes	7	6	3

### 4.2. Baselines

We compare our MT-GAT against the following baselines: graph convolution networks (GCN), graph autoencoder (GAE, VGAE), graph attention networks (GAT), local neighborhood graph autoencoder (LoNGAE, αLoNGAE).

- GCN (Kipf and Welling, 2017) performs a convolution operation on each node's neighbors for feature aggregation in each graph convolutional layer, which can be stacked to deeper networks for semi-supervised node classification tasks.
- GAE and VGAE (Kipf and Welling, 2016) utilize a graph convolutional network (GCN) encoder and a simple inner product decoder. The advantage of this method is that it can naturally incorporate node features compared to most existing unsupervised models for link prediction.
- GAT (Veličković et al., 2018) is a novel neural network architecture that operates on graph-structured data, leveraging masked self-attentional layers to address the shortcomings of prior methods based on graph convolution or their approximation.
- LoNGAE and αLoNGAE (Tran, 2018) introduce a densely connected autoencoder architecture to learn a joint representation of both local graph structure and available external node features for the multi-task learning of link prediction and node classification. LoNGAE and αLoNGAE adopt the densely connected symmetrical autoencoder, where αLoNGAE uses node features and LoNGAE does not. In our node classification experiments, we only adopt αLoNGAE for comparison due to its superiority.

### 4.3. Experimental Settings

We implement our MT-GAT with the Pytorch-GPU backend, along with several additional details. Gradient descent optimization is employed with a fixed learning rate of 0.005. Two layers of dropout are used in the model with dropout rate of 0.1 to prevent the problem of overfitting. The number of attention heads in the graph attention layer is set to 8, consistent with the setting for transductive learning in GAT. We train for 300 epochs for MT-GAT. The loss of node classification is negative log likelihood loss while the loss of link prediction is binary cross entropy. The tradeoff factor between node classification and link prediction tasks α is 1. For fair comparison, we use mean classification accuracy to measure the performance of the node classification task, and use AUC and AP to evaluate the results of link prediction. The evaluation metric AUC is the area under the ROC curve. In the context of unbalanced categories, even if the number of certain categories increases significantly, the growth of the curve is not obvious, and therefore we choose it to eliminate the impact of a lot of imbalanced classes. AP is just the average accuracy score.

### 4.4. Results and Analysis

We use different methods to obtain embedding vectors of nodes, and adopt softmax as classifier. For comparison, the training ratio of the classifier is ranged from 10 to 90% with a step of 10% in each dataset for all methods. We run each method 10 times, respectively at a given training ratio and report the average performance.

Tables 3–5 demonstrate the comparison of mean classification accuracy on semi-supervised node classification for GCN, αLoNGAE, GAT, and our MT-GAT. For clarity, the best results are shown in bold. For node classification, GCN and our MT-GAT exhibit better performance compared with LoNGAE and GAT. Although GCN occasionally outperforms our MT-GAT on the Pubmed dataset when the training ratio is 90%, it is inferior to our MT-GAT in all other cases. It is shown that on this task, the performance of our MT-GAT is relatively stable and splendid compared with baselines, which fully demonstrates the superiority of our multi-task network representation learning framework. Furthermore, we conduct the *t*-test in Tables 3–5 and our MT-GAT with significant improvements over the baselines is shown with underline as measured by a *t*-test with a *p*-value ⩽ 0.05.

**Table 3:** Accuracy of semi-supervised node classification on Cora.

**Method**	**90%**	**80%**	**70%**	**60%**	**50%**	**40%**	**30%**	**20%**	**10%**
GCN	0.842	0.842	0.828	0.828	0.821	0.821	0.807	0.807	0.800
αLoNGAE	0.803	0.793	0.790	0.783	0.780	0.777	0.770	0.767	0.763
GAT	0.824	0.822	0.816	0.808	0.806	0.804	0.798	0.796	0.794
MT-GAT (ours)	**0.874**	**0.864**	**0.861**	**0.856**	**0.855**	**0.850**	**0.848**	**0.832**	**0.827**

*The best results are shown in bold, and our MT-GAT with significant improvements over the baselines is shown with underlines*.

**Table 4:** Accuracy of semi-supervised node classification on Citeseer.

**Method**	**90%**	**80%**	**70%**	**60%**	**50%**	**40%**	**30%**	**20%**	**10%**
GCN	0.846	0.824	0.824	0.824	0.813	0.802	0.802	0.780	**0.780**
αLoNGAE	0.733	0.727	0.723	0.716	0.710	0.706	0.697	0.690	0.683
GAT	0.718	0.716	0.710	0.708	0.706	0.704	0.700	0.698	0.696
MT-GAT (ours)	**0.852**	**0.845**	**0.841**	**0.835**	**0.830**	**0.820**	**0.816**	**0.800**	**0.780**

*The best results are shown in bold, and our MT-GAT with significant improvements over the baselines is shown with underlines*.

**Table 5:** Accuracy of semi-supervised node classification on Pubmed.

**Method**	**90%**	**80%**	**70%**	**60%**	**50%**	**40%**	**30%**	**20%**	**10%**
GCN	**0.871**	0.838	0.838	0.806	0.806	0.774	0.774	0.741	0.741
αLoNGAE	0.807	0.803	0.800	0.797	0.796	0.793	0.790	0.787	0.786
GAT	0.794	0.792	0.790	0.788	0.786	0.784	0.782	0.780	0.788
MT-GAT (ours)	0.854	**0.847**	**0.843**	**0.836**	**0.831**	**0.824**	**0.822**	**0.816**	**0.806**

*The best results are shown in bold, and our MT-GAT with significant improvements over the baselines is shown with underlines*.

Table 6 shows the comparison of AUC and AP performance on link prediction for GAE, VGAE, LoNGAE, αLoNGAE, GCN, and MT-GAT. For link prediction, the LoNGAE that only captures graph structure without node features is less than satisfactory, but the αLoNGAE with node features performs slightly better. Although αLoNGAE occasionally outperforms our MT-GAT on the Cora and Citeseer datasets, αLoNGAE is restrictive and obviously provides no flexibility in extending to general network representation learning methods. In the meantime, the performance of GAE and VGAE is mediocre because it is potentially a poor choice in combination with an inner product decoder, and the generative model is not flexible enough. Note that in this task, our MT-GAT performs comparable or more excellent than other methods, due to the capability of our framework for collaboratively learning task-oriented embedding representations.

**Table 6:** AUC and AP performance of different methods on link prediction.

**Method**	**Cora**		**Citeseer**		**Pubmed**	
	**AUC**	**AP**	**AUC**	**AP**	**AUC**	**AP**
GAE	0.910	0.920	0.895	0.899	0.964	0.965
VGAE	0.914	0.926	0.908	0.920	0.944	0.947
LoNGAE	0.896	0.915	0.860	0.892	0.926	0.930
αLoNGAE	**0.943**	0.952	**0.956**	**0.964**	0.960	0.963
GCN	0.809	0.811	0.811	0.822	0.828	0.834
MT-GAT (ours)	0.930	**0.963**	0.931	0.963	**0.968**	**0.970**

*The best results are shown in bold*.

Overall, our MT-GAT achieves more outstanding and stable performance on both tasks of node classification and link prediction. However, these baselines mostly learn network representations based on a model-dependent framework without careful consideration of the follow-up tasks to optimize the embedding model. Our MT-GAT is simultaneously supervised by node classification and link prediction tasks, and is capable of learning comprehensive and desirable node representations. Through the joint learning of two different loss functions, our model is able to achieve more effective, complete, and stable predictions.

### 4.5. Parameter Sensitivity

**Figure 3:**
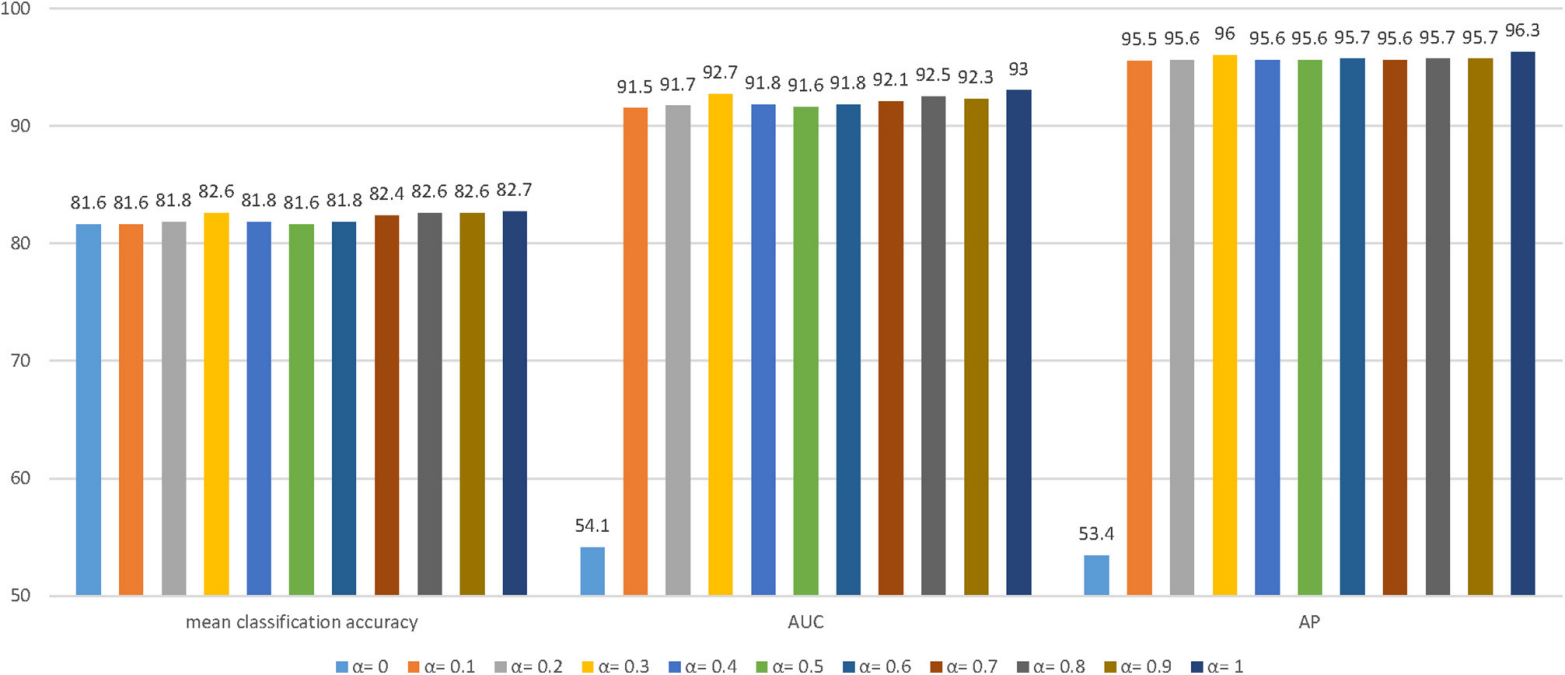
The effect of different hyperparameters α on the Cora dataset. We choose the AUC and AP scores of link prediction and the classification accuracy of node classification to demonstrate the effect of different hyperparameters for the experiments.

The parameter sensitivity of MT-GAT is investigated in this section. More specifically, we evaluate how different values of hyperparameter α can affect the performance of node classification and link prediction. The hyperparameter α is varied from 0 to 1 with an increment of 0.1. We report the three evaluation metrics: mean classification accuracy for node classification, AUC score for link prediction, and AP scores for link prediction. The histogram in Figure 3 displays the results of evaluation metrics with different parameter settings for the Cora dataset. We notice that the performance of node classification and link prediction on the Cora dataset fluctuates from α = 0 to 1. It slightly boosts at first and reaches the local optimum at α = 0.3. After the value of α is over 0.3, it gradually declines and slightly increases to the peak at α = 1. The AUC and AP scores of link prediction are more sensitive to parameters than the classification accuracy of node classification. Especially, when parameter α is 0, the optimization of the link prediction loss is completely separated from that of the network embedding model, thus causing AUC and AP scores of link prediction to always float around the starting value of 0.5. It empirically suggests that the consideration of the weight parameter α between node classification and link prediction tasks can facilitate learning network representations more effectively.

## 5. Conclusion

In this paper, we propose a multi-task network representation learning framework, namely MTNRL, which exploits the synergy among the node classification and link prediction tasks for facilitating their individual performance. The experimental results demonstrate the MTNRL framework on GAT is well-performed on a range of graph-structured network datasets for both node classification and link prediction. Besides, the proposed method can soundly outperform the state-of-the-art network representation learning methods. The main advantage of our MT-GAT is the performance improvement brought by the extensive parameter sharing between link prediction and node classification tasks. The proposed framework solves the single-task limitations of traditional network representation learning methods. In particular, our framework is universal and can be implemented on any arbitrary network embedding methods to improve performance. In future work, we will investigate the implementation of our framework on heterogeneous network representation methods and explore the scalability of our framework on other network analysis tasks.

## Data Availability Statement

The datasets analyzed in this manuscript are not publicly available. Requests to access the datasets should be directed to peixuanjin@gmail.com.

## Author Contributions

YX and PJ conceptualized the problem and the technical framework. MG and CZ developed the algorithms, supervised the experiments, and exported the data. YX, PJ, and BY implemented the multi-task representation learning architecture simulation. BY managed the project. All authors wrote the manuscript, discussed the experimental results, and commented on the manuscript.

### Conflict of Interest

The authors declare that the research was conducted in the absence of any commercial or financial relationships that could be construed as a potential conflict of interest.
